# Gait Symmetry Assessment with a Low Back 3D Accelerometer in Post-Stroke Patients

**DOI:** 10.3390/s18103322

**Published:** 2018-10-03

**Authors:** Wei Zhang, Matthew Smuck, Catherine Legault, Ma A. Ith, Amir Muaremi, Kamiar Aminian

**Affiliations:** 1 Laboratory of Movement Analysis and Measurement Ecole Polytechnique Fédérale de Lausanne (EPFL), 1015 Lausanne, Switzerland; kamiar.aminian@epfl.ch; 2 Wearable Health Lab, Department of Orthopaedic Surgery, Stanford University, Redwood City, CA 94063, USA; msmuck@stanford.edu (M.S.); mith@stanford.edu (M.A.I.); 3 Division of Physical Medicine & Rehabilitation, Stanford University, Palo Alto, CA 94304, USA; 4 Stanford Stroke Center, Stanford University, Palo Alto, CA 94304, USA; clegault@stanford.edu; 5 Novartis Institutes for BioMedical Research, 4056 Basel, Switzerland; amir.muaremi@novartis.com

**Keywords:** symmetry, trunk movement, autocorrelation, gait rehabilitation

## Abstract

Gait asymmetry is an important marker of mobility impairment post stroke. This study proposes a new gait symmetry index (GSI) to quantify gait symmetry with one 3D accelerometer at L3 (GSI_L3_). GSI_L3_ was evaluated with 16 post stroke patients and nine healthy controls in the Six-Minute-Walk-Test (6-MWT). Discriminative power was evaluated with Wilcoxon test and the effect size (ES) was computed with Cliff’s Delta. GSI_L3_ estimated during the entire 6-MWT and during a short segment straight walk (GSI_L3straight_) have comparable effect size to one another (ES = 0.89, *p* < 0.001) and to the symmetry indices derived from feet sensors (|ES| = [0.22, 0.89]). Furthermore, while none of the indices derived from feet sensors showed significant differences between post stroke patients walking with a cane compared to those able to walk without, GSI_L3_ was able to discriminate between these two groups with a significantly lower value in the group using a cane (ES = 0.70, *p* = 0.02). In addition, GSI_L3_ was strongly associated with several symmetry indices measured by feet sensors during the straight walking cycles (Spearman correlation: |ρ| = [0.82, 0.88], *p* < 0.05). The proposed index can be a reliable and cost-efficient post stroke gait symmetry assessment with implications for research and clinical practice.

## 1. Introduction

Stroke is the fifth leading cause of death in the United States [1]. About 80% of stroke survivors are affected by hemiparesis [1], characterized by muscle weakness and extensor spasticity in the lower extremities that can severely influence mobility in post-stroke patients. One of the typical impairments caused by this hemiparesis is gait asymmetry. In post-stroke rehabilitation programs, considerable focus is placed on the equalization of weight bearing through the lower extremities and the capacity to shift weight between the lower extremities during gait [2]. Symmetry is a target gait function to restore and is an useful outcome measure of the rehabilitation [2,3,4,5,6]. Despite its clinical interest and importance, there is currently no standardized method to measure gait symmetry [6,7]. Camera-based motion capture system, and pressure mats and insoles are used to analyze kinetics and kinematics of gait [4,6,8,9,10], and fewer studies reported different methods for gait symmetry assessment using inertial sensors [10,11,12]. Regardless of the tool used to measure gait, the assessment of symmetry remains a simple comparison of different spatiotemporal gait parameters between left and right sides (such as stance and swing phase, step length, etc.). To our knowledge, only one study has reported an alternative method other than aforementioned studies based on spatiotemporal gait parameters to analyze symmetry [13]. Using inertial sensors fixed on each shank, the method first applied quantization of raw sensor signals with a symbolic segmentation, then symmetry is determined by the difference ratio between the symbols of left and right side. However, a user-defined threshold on the mean squared error to determine the quantization resolution a priori limits this method’s accuracy. In a recent study, we developed two simple gait symmetry indices estimated by the linear correlation coefficient and the normalized sample distance between the left and right foot pitch angular velocity [14]. The developed indices demonstrated high and comparable discriminative power than gait symmetry estimated with spatiotemporal gait parameters. Importantly, these novel methods assess gait symmetry based on signal profiles corresponding to step alternation from two sides without estimation of specific spatiotemporal gait parameters, which usually requires sophisticated signal processing and a gait model. Such a gait model is usually developed for healthy gait patterns and its reliability becomes questionable in applications with pathological gait.

Analyzing step alternation from sensor signal profile might be applicable to other sensor configurations as well. For example, one study [12] reported that asymmetry estimated by the trunk movement showed significant difference between chronic stroke patients and healthy controls, whereas comparison of side-to-side symmetry in spatiotemporal parameters did not differ between groups. However, the study reported a comparison of symmetry outcomes with only two spatiotemporal gait patterns. It is not fully clear whether the trunk movement is superior to feet kinematics for gait asymmetry estimation in post stroke patients.

In this study, we proposed and evaluated a gait symmetry index derived from a single 3D accelerometer worn at the midline of the lower back (approximately at the level of L3). The primary outcome of this study was the discriminative power of the single low back accelerometry based gait symmetry index in post-stroke patients. We compared the discriminative power to the gait symmetry indices estimated using two feet sensors that have been validated in previous studies, namely the method based on the difference ratio of various spatiotemporal gait parameters and the method based on the pitch angular velocity signal profile. The secondary outcome was the association between gait symmetry estimated by one low back accelerometer and the symmetry measured with two feet sensors.

## 2. Materials and Methods

### 2.1. Data Acquisition

Sixteen consecutively consenting post-stroke patients (nine males and seven females, average age 54 years with range 23–74, 6 using a cane) and nine healthy controls (five males and four females, average age 35 years with range 25–48) participated this study at the Physical Medicine & Rehabilitations Section and in the Department of Neurology & Neurological Sciences, at Stanford University. Six patients suffered from subcortical stroke and 10 suffered from cortical stroke. The stroke etiology was hemorrhagic in one patient and ischemic in the others. The time after stroke varied from 5 months to 11 years with a median of 20 months. Patient self-reported outcome of stroke impact scale (SIS) [15] was between 190 to 288 points with a median of 219.5 (the higher the point, the higher the impact). The ethical committee for Human Subjects Research at Stanford University approved this study. Written informed consents were obtained from the patients in the study. 

Participants performed gait assessment in Six-Minute-Walk-test (6-MWT) with their comfortable walking speed under the supervision of an experienced clinical researcher. The 6-MWT is a reliable and easy to administer assessment widely used for walking function assessment following stroke [16]. At our location, the 6-MWT was conducted in a rectangular set of corridors between 15 to 25 m long linked together by 90-degree turns. During the test, patients wore a wireless inertial sensor (MTw Awinda, Xsens, Enschede, The Netherlands [17]) on top of each shoe and in the midline of the low back (approximately at the level of L3). Each sensor was held in position with an elastic band. Study participants were provided instruction before the walking assessment. When ready at the starting position (standing straight in front of the starting line), data recording started with the countdown 5 seconds prior to the command ‘Start’ from the clinical researcher. Recording stopped when the participant completed the 6-MWT and stood quietly at the end of the trial. Raw sensor data were transmitted to a Windows laptop via Bluetooth during the assessment. To assure good connection, a researcher walked with the laptop behind the participants during the entire 6-MWT. Meanwhile, the researcher marked down the time when the participants reached the first 90-degree turn at the end of the corridor (ca. 14 m). The sensor data collection application running on the laptop synchronized the data and exported a data file for each assessment containing 3D acceleration and 3D gyroscope data sampled at 100 Hz. 

### 2.2. Gait Symmetry Assessment with Two Feet Sensors

#### 2.2.1. Symmetry of Spatiotemporal Gait Parameters

We used validated algorithms [18,19,20,21] to extract spatiotemporal gait parameters from the feet sensors. Prior to processing, data were resampled to 200 Hz using linear interpolation to be consistent with the validated algorithms. In specific, gait cycles were detected based on the timing of two consecutive foot-flats [19]. Velocity and position of the foot were estimated by the numerical integration of the gravity-free acceleration data in the global frame and drift removal technique using the zero velocity update during the foot-flat period [22]. Subsequently, path length, ratio between the actual 3D path and the stride length, were estimated [22]. Heel strike and lift off angles were estimated based on the de-drifted angular velocity data [21]. Maximum angular velocity of the foot and various temporal parameters were extracted from the angular velocity signals [19]. Cycles with a turning angle between two foot-flats less than 20 degrees were considered as straight walking cycles [18]. Symmetry index (SI) was estimated using the difference ratio of the spatiotemporal parameters (listed in Table 1) of each gait cycle *n* according to Equation (1):

(1) $$\mathrm{SI}\left(n\right)=\frac{Param_{left}\left(n\right)-Param_{right}\left(n\right)}{0.5*\left[Param_{left}\left(n\right)+Param_{right}\left(n\right)\right]}*100\%$$

#### 2.2.2. Symmetry of Foot Pitch Angular Velocity

Gait symmetry was computed using the recently published algorithms [14]. The algorithms assessed symmetry using the foot pitch angular velocity signals of each gait cycle. The pitch angular velocity signal was smoothed with a 2nd order Butterworth low-pass filter (cut-off frequency of 10 Hz). The maximum lag between the signals from both feet was estimated based on cross correlation, and one signal was shifted to align left and right gait cycles. The aligned signals were segmented to individual gait cycles based on detected gait cycle of the right foot leading to two signals ω*_left_*(*n*) and ω*_right_*(*n*) of cycle *n* [19]. The gait symmetry between the left and the right signals was assessed for each cycle based on (a) Pearson correlation coefficient (denoted by *GSI_corr_*) and (b) the normalized sample distance (denoted by *GSI_dist_*). *GSI_dist_* was the mean absolute difference between each left and right signal sample of cycle *n* divided by the mean range of the signals in the cycle (Equation (2)). Mean values of *GSI_corr_* and *GSI_dist_* of all straight walking cycles in the entire 6-MWT were calculated. The detection of gait cycle and the selection of straight walking cycles were based on the same algorithms mentioned in Section 2.2.1:

(2) $$GSI_{dist}\left(n\right)=\frac{mean\left(\left|\omega_{left}\left(n\right)-\omega_{right}\left(n\right)\right|\right)}{0.5*\left[range\left(\omega_{left}\left(n\right)\right)+range\left(\omega_{right}\left(n\right)\right)\right]}*100\%$$

### 2.3. Gait Symmetry Assessment with a Single 3D Accelerometer at the Low Back

Gait cycles can be measured by analyzing the repetitive movement pattern of the center of mass (CoM) [23]. Low back (approximately L3) accelerations, which are assumed to correspond to CoM during walking, were first smoothed with a 2nd order Butterworth low-pass filter with the cut-off frequency of 10 Hz. Autocorrelation coefficients of vertical (*AR_v_*), frontal (*AR_f_*) and lateral (*AR_l_*) accelerations at the low back were computed as the function of time lag (*t*), respectively. The biased form of autocorrelation was used to suppress the amplitude of the coefficients while *t* increased [23]. The maximum time lag was 4 s (400 samples), which is about 2.5 times a single stride duration in post hemiplegic stroke patients [24]. This window length was chosen to capture the repetition of stride cycles in very slow walking. Coefficient of stride cycle repetition (C_stride_) was the sum of positive autocorrelation coefficients of the three axes as a function of *t* (Equation (3)). Coefficient of step repetition (C_step_) was the norm of autocorrelation coefficients as a function of *t* (Equation (4)). One stride time (*T_stride_*) equals to *t*, when C_stride_ had the maximum value. The hypothesis was that, in a perfect symmetric gait pattern, two consecutive steps have the same step duration of 0.5 * *T_stride_*. The maximum value of C_step_ was $\sqrt{3}$ when autocorrelation coefficient of each acceleration axis was 1 at zero-lag (*t* = 0). The gait symmetry index (GSI_L3_) was C_step_ (0.5 * *T_stride_*) normalized to its value at zero-lag (Equation (5)), so that the maximum value of GSI_L3_ was 1 in a perfect symmetric gait pattern:

(3) $$C_{\mathrm{stride}}(t)=AR_{v}\left(t\right)+AR_{f}\left(t\right)+AR_{l}\left(t\right);\;\mathrm{if}\;AR\left(t\right)<0,\;AR\left(t\right)=0$$

(4) $$C_{\mathrm{step}}(t)=\sqrt[{2}]{AR_{v}\left(t\right)+AR_{f}\left(t\right)+AR_{l}\left(t\right)}$$

(5) $${\mathrm{GSI}}_{L3}=C_{\mathrm{step}}\;(0.5*T_{stride})/\sqrt{3}$$

### 2.4. Statistical Analysis

Symmetry indices (SI) of each spatiotemporal gait parameter estimated by the feet sensors listed in Table 1, *GSI_corr_*, *GSI_dist_* and GSI_L3_ (estimated by the low back accelerometer) were computed for each post-stroke patient and each healthy control. For SI, *GSI_corr_* and *GSI_dist_*, mean values over all gait cycles during straight walking in the entire 6-MWT assessment period were computed. GSI_L3_ was computed for the entire 6-MWT assessment period and for the first straight course of the assessment (GSI_L3straight_). Given the small sample size in this study, non-parametric statistics were applied for the analyses. Wilcoxon rank sum test was used to test whether there are significant differences in various sensor-derived gait symmetry indices between post-stroke patients and control group. In addition, effect size (ES) calculator Cliff’s Delta was used to determine the discriminating power of various symmetry indices [25]. Cliff’s Delta calculates the proportion of non-overlapped samples in the groups. ES = 1 or −1 indicates the two groups have no overlap. Whereas, ES = 0 means the two groups are not separable. According to a study by Romano et al., ES less than 0.147 is negligible, between 0.147 and 0.33 is small, between 0.33 and 0.474 is medium, and more than 0.474 is a large effect [26]. The correlations between the low back sensor derived symmetry indices (GSI_L3_ and GSI_L3straight_) and the feet sensor based symmetry indices (SI, *GSI_corr_* and *GSI_dist_*) were analyzed with Spearman rank correlation coefficient (ρ).

## 3. Results

### 3.1. Discriminative Power of Gait Symmetry as Measured by Various Indices

**Figure 1:**
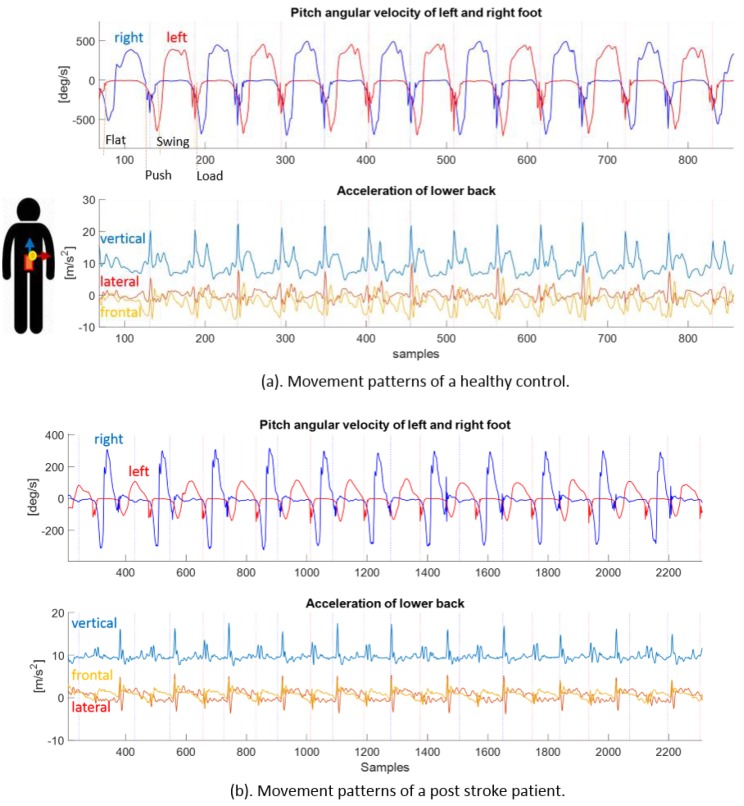
Synchronized pitch angular velocity signals from feet sensors and 3D acceleration signals from low back sensor. (**a**) Synchronized signals from a healthy control. (**b**) Synchronized signals from a post-stroke patient. Upper plot shows foot pitch angular velocity on the left (red) and right (blue) side during walking. Lower plot shows lower back acceleration on the vertical (blue), frontal (yellow) and lateral (red) axis. The dotted vertical lines indicate of each gait cycles detected by the feet sensors. In (**a**), time phases of foot-flat, push-up, swing and loading in one cycle of the left foot are indicated in the pitch angular velocity signal. Axes of the accelerometer at the low back are illustrated next to the acceleration signals.

Comparison between the synchronized feet pitch angular velocity signals and the low back acceleration signals revealed that the CoM movement repeated with the gait cycles. Figure 1a shows the sensor signals of a healthy control. The vertical acceleration showed stronger repetitive patterns than frontal and lateral accelerations at each step corresponding to the foot pitch angular velocity of left and right steps, which had high similarity in this healthy control. Whereas, the lateral acceleration shows a strong repetitive pattern with each stride (two steps). 

**Figure 2:**
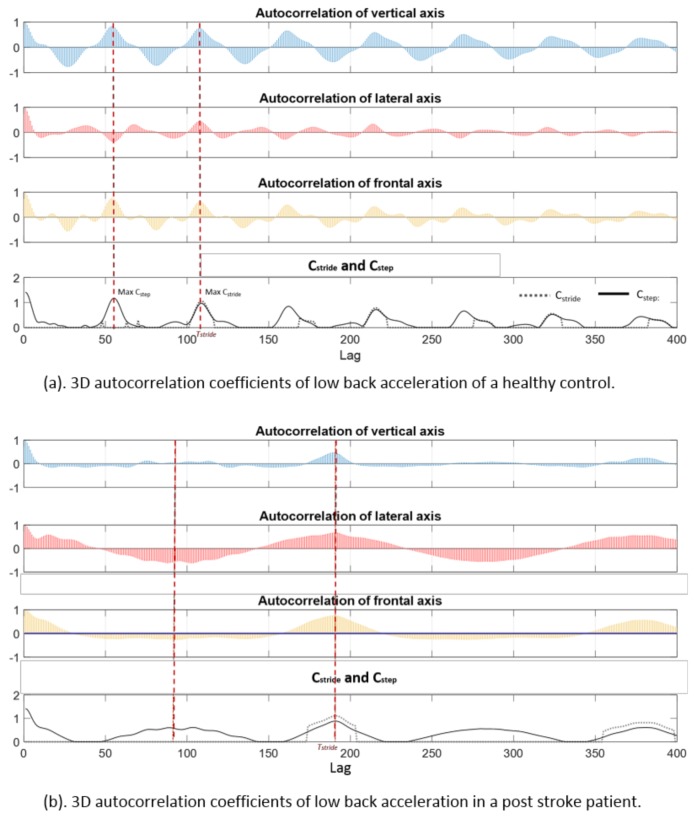
Autocorrelation coefficients of 3D acceleration of lower back. (**a**) Coefficients of a healthy control. (**b**) Coefficients of a post-stroke patient. Autocorrelation coefficients in vertical (blue), lateral (red) and frontal (yellow) axis are computed with increased lag from 0 to 400 samples (4 s). C_stride_ (dotted black line) and C_step_ (solid black line) in the bottom plot are computed as a function of time lag.

Figure 1b shows sensor signals of a post-stroke patient. The foot pitch angular velocity profiles between the left and right steps had visible differences, which were reflected in the movement of the CoM as well. The vertical acceleration at the low back had poor similarity between the successive steps, but was visible between successive stride cycles. The aforementioned signal patterns were captured by the autocorrelation coefficients, C_stride_ and C_step_ as illustrated in Figure 2. The healthy control (a) had a shorter stride duration (ca. 1.05 s at maximum C_stride_) compared to the post-stroke patient (b) (ca. 1.90 s). The coefficient of step repetition (C_step_) of the healthy control at half stride time was higher than that in the post-stroke patient, which indicated a higher gait symmetry. 

**Figure 3:**
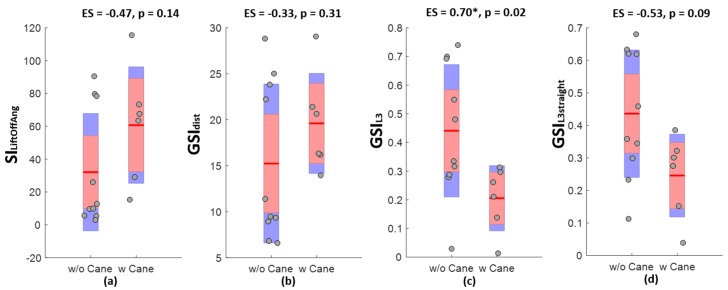
Boxplots of various gait symmetry indices measured in post-stroke patients with (w Cane) or without (w/o Cane) using cane. Effect size (ES) is measured with Cliff’s Delta and *p* value is determined by Wilcoxon rank sum test. * indicates *p* < 0.05. (**a**) Comparison and effect size of SI_LiftOffAng_. (**b**) Comparison and effect size of *GSI_dist_*. (**c**) Comparison and effect size of GSI_L3_. (**d**) Comparison and effect size of GSI_L3straight_.

Table 2 summaries the discriminative power of various gait symmetry indices. Gait symmetry measured with two feet sensors demonstrated a significant difference between healthy controls and post-stroke patients (except for symmetry of foot loading and flat ratios), among which, *GSI_dist_* had the largest effect size. Gait symmetry measured with the low back accelerometer was significantly lower in post-stroke patients during the entire 6-MWT and during the shorter straight walk of the assessment. The effect size was the same as *GSI_dist_*, the best spatiotemporal parameter derived from the feet angular velocity signals. Boxplots in Figure 3 show the differences in various symmetry indices between post-stroke patients walking with and without a cane. Interestingly, GSI_L3_ was significantly lower in post-stroke patients walking with a cane compared to those able to walk without. There were no significant differences between GSI_L3straight_ and any symmetry estimates provided by the feet sensors. 

### 3.2. Correlations between Gait Symmetry Measured with Low Back Accelerometry and That Measured with Two Feet Sensors

**Figure 4:**
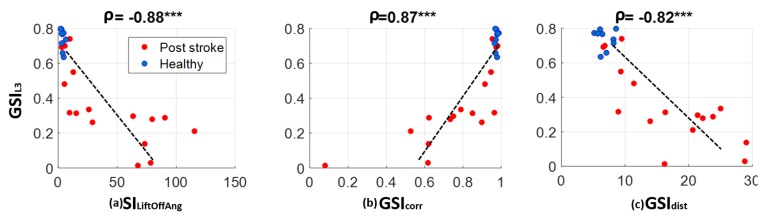
Correlation between gait symmetry measured with the low back accelerometer and symmetry measured with two feet sensors. Association is estimated with Spearman correlation. *** indicates *p* < 0.0001.

Correlation analysis shown in Figure 4 indicated good consistency between gait symmetry measured with single 3D accelerometer at the low back and those measured with two feet sensors during the straight walking cycles of entire 6-MWT (ρ = −0.88 with SI_LiftOffAng,_ ρ = 0.87 with SI_corr_ and ρ = −0.82 with *GSI_dist_*). Gait symmetry derived from the low back accelerometer when the participants walked through a short straight path (GSI_L3straight_) were significantly correlated with feet sensor based symmetry measures as well (ρ = −0.84 with SI_LiftOffAng,_ ρ = 0.80 with GSI_corr_ and ρ = −0.79 with *GSI_dist_*). 

## 4. Discussion

This study developed a gait symmetry assessment with a single 3D accelerometer placed at the low back. Symmetry index estimated with the low back accelerometer, GSI_L3_, is a measure of the repetitiveness of the gait cycles. Thus, the more symmetric the gait is, the higher the index value. On the contrary, the symmetry indices based on the spatiotemporal gait parameters with the feet sensors, SI, are measures of the degree of difference in the bilateral movement. The value decreases when the difference decreases as in symmetric gaits. Thus, SI has a negative correlation with GSI_L3_. This is also the case for the gait symmetry measured with angular velocity signal profile *GSI_dist_* using the feet sensors, as *GSI_dist_* measures the difference between the bilateral foot angular velocity signals. GSI_corr_ with the feet sensors measures the correlation between the bilateral foot angular velocity signals. Its value increase when signals have higher correlation as in symmetric gaits. Hence, GSI_corr_ has a positive correlation with GSI_L3_. More importantly, GSI_L3_ has good discriminative power comparable to symmetry indices based on spatiotemporal parameters derived from two feet sensors. GSI_L3_ has several advantages in technical implementation and clinical practice. 

### 4.1. Advantages in Technical Implementation

A quantitative measure of the degree of asymmetry is useful for post-stroke gait rehabilitation assessment. Computing difference ratio of left and right steps based on spatiotemporal foot characteristics processes accelerometer, gyroscope, and in some cases magnetometer, barometer and foot pressure data [27], to derive gait parameters, which are high-level descriptions of information contained in the raw sensor signals. Gait modeling, advanced signal processing and complex 3D computation are required to find accurate spatiotemporal measures [18,19,20,21]. However, the challenge of accurate gait parameter estimation rises when applying the model to different pathologies, which can deviate largely from normal gait patterns. Often observed in post-stroke patients, lower limb movement is impaired by stiffness and slowness, which imposes difficulty to estimates of displacement, speed or rotation in periodic movement based on integration of inertial sensor signals [28]. Thus, the reliability of spatiotemporal-derived parameters may become questionable. To avoid this, computing symmetry (GSI_L3_) is based on analysis of acceleration signals’ repetitiveness quantified by autocorrelation coefficients. In addition, the computation is both easier and more robust than the morphology-based signal processing provided by the spatiotemporal gait parameter estimations. Compared to symmetry indices estimated with two feet sensors, GSI_L3_ is easier for technical implementation as only a single sensor is required. The computation of GSI_L3_ is based on the norm of autocorrelation coefficients rather than analysis of individual axis as presented in two studies [12,23]. Different from these studies, the proposed estimation of GSI_L3_ does not rely on detection of step alternation, which can be unreliable in people with poorly symmetric gaits as shown in Figure 2b. The biased autocorrelation coefficients decreases while time lag increases, which allows the reliable detection of the immediate next stride. In addition, estimation of GSI_L3_ during the entire 6-MWT as presented in this study has comparable discriminative power as those estimations using cleaned data (only straight walking cycles) with two feet sensors as shown in the results summarized in Table 2. Ultimately, GSI_L3_ requires less computation and it may be more feasible and robust than feet sensor based gait symmetry measures in semi- or unsupervised assessment. 

### 4.2. Advantages in Clinical Practice

Gait symmetry is a biomarker of post-stroke rehabilitation [4]. Compared to assessment with two feet sensors, use of a single sensor worn at the low back is easier to set up in an office setting and less prone to disruption. Our results show that the low back sensor symmetry index estimated with a short straight walk is similar to that estimated with a complete 6-MWT. This implies that further simplification of the current clinical assessment procedure, to a brief walking assessment is possible. Asymmetry in spatiotemporal gait characteristics of post-stroke population, such as stance ratio, has been confirmed in other studies [3,29]. In this study, we demonstrate that asymmetry measure with a single low back accelerometer can significantly differentiate post-stroke gait from healthy gait, and can do so with an effect size that is larger than the difference in stance ratio and comparable to the best performing spatiotemporal characteristics (path length, maximum angular velocity and angle at toe lift off). This finding is confirmed by other studies of stroke survivors, where repetitiveness of the trunk accelerometry performed better than similarity between left and right step length and stride duration [12]. Furthermore, the developed symmetry index can discriminate severity of gait disturbance in the post-stroke population. In our study, the low back symmetry index could discriminate between gaits of post-stroke patients that required a cane from those who ambulating without an assistive device, comparing to the symmetry indices derived from two feet sensors that did not show significant difference between these two groups. These results suggest that the CoM accelerometry from the single low back sensor might be more sensitive to disability severity than feet kinematics within post-stroke patients. Accordingly, we suggest that gait symmetry assessment with one sensor on the low back is preferred over assessments with two feet sensors, both for in-clinic assessments and for long-term unsupervised assessments outside the clinic setting. 

### 4.3. Limitations

The presented analyses have some limitations. We did not calibrate the low back accelerometer before gait symmetry estimation. Removal of static offset in acceleration signals and accurate alignment of axes with trunk frame may improve reproducibility of the symmetry index estimation. This is particularly important for gait symmetry assessment in individual patients during rehabilitation. In addition, test-retest reliability of the presented GSI_L3_ should be evaluated to determine the minimum detectable change using the developed index. Another limitation is with the selection of the maximum time lag for autocorrelation analysis. Four-second lag was chosen based on reported data in previous stroke study. A longer window is unlikely to affect the stride time detection. However, a shorter window may not accurately detect the stride repetition in extremely impaired stroke patients with very slow walking. A systematic examination using different window length will be required to determine the optimized configuration for computation with a patient group exhibiting large functional variations. In addition, the sample size in this study was small, yet our findings did reach statistical significance with effect sizes that suggest the sample was sufficient to support our conclusion. Still, it must be noted that age differences between the post-stroke and the control group may introduce some bias into the effect sizes of the various symmetry indices. 

### 4.4. Future Studies

In the on-going study, we will address the clinical relevance of the developed gait symmetry assessment. Associations between clinical stroke diagnosis (including stroke etiology, SIS, self-reported recovery assessment) and the developed gait symmetry index will be analyzed. The analysis outcome will be compared to that measured by other more traditional gait markers, such as gait speed. The test-retest reliability of the developed gait symmetry index will be studied to determine the minimum detectable change that is relevant for longitudinal gait rehabilitation assessment. Future studies will also test the proposed symmetry index with large sample size to confirm the results of this study. 

## 5. Conclusions

The proposed gait symmetry assessment with one 3D accelerometer placed on the midline of the low back shows high discriminative power in differentiating post-stroke patients from healthy controls. The outcome is comparable to the gait symmetry with spatiotemporal gait analysis using two feet sensors. The proposed method can be a cost-effective and reliable solution for post-stroke gait symmetry assessment in clinic. Assessment reproducibility and feasibility for unsupervised assessment is an important next step in our investigation to enable future studies using this method to monitor gait recovery after stroke, and to guide post-stroke gait rehabilitation. 

## Figures and Tables

**Table 1:** Spatiotemporal parameters analyzed for each foot in one gait cycle.

Parameter [Unit]	Description
**Spatial**	
PathLength [% stride length]	Ratio between the length of the real path of the foot in 3D space (including both stride length and width) and stride length of one cycle.
StrikeAng [deg]	Angle between the foot and the ground at heel strike in sagittal plane.
LiftOffAng [deg]	Angle between the foot and the ground at toe off in sagittal plane.
MaxAngVel [deg/s]	Maximum pitch foot angular velocity during swing phase.
**Temporal**	
StanceRatio [%]	Percentage of the gait cycle during which the foot is in stance phase.
LoadRatio [%]	Percentage of the stance corresponding to loading phase defined as the time between heel strike and toe strike
FootFlatRatio [%]	Percentage of the stance corresponding to the foot-flat phase
PushRatio [%]	Percentage of the stance corresponding to push phase defined as the time between heel off and toe off.

**Table 2:** Mean ± standard deviation and effect size (ES) as estimated by Cliff’s Delta of various symmetry indices.

Symmetry Index (SI)	Control Group	Post-stroke	ES
**Gait symmetry based on spatiotemporal gait parameter**			
**PathLength**	0.54 ± 0.07	4.30 ± 5.69	−0.85 ***
**StrikeAng**	8.50 ± 4.53	35.78 ± 30.85	−0.79 **
**LiftOffAng**	3.78 ± 1.33	42.80 ± 37.27	−0.88 ***
**MaxAngVel**	6.31 ± 3.78	44.86 ± 39.23	−0.81 **
**StanceRatio**	3.06 ± 2.27	12.19 ± 8.06	−0.79 **
**LoadRatio**	22.36 ± 9.42	32.36 ± 22.06	−0.22
**FootFlatRatio**	5.99 ± 2.28	7.18 ± 4.98	−0.13
**PushRatio**	6.88 ± 3.67	25.76 ± 25.57	−0.71 **
**Gait symmetry based on feet angular velocity signal profile**			
$GSI_{corr}$	0.97 ± 0.01	0.76 ± 0.24	0.85 ***
$GSI_{dist}$	6.84 ± 1.23	16.87 ± 7.70	−0.89 ***
**Gait symmetry based on low back accelerometry**			
$GSI_{L3}$	0.74 ± 0.06	0.35 ± 0.22	0.89 ***
**$GSI_{L3straight}$**	0.69 ± 0.09	0.36 ± 0.19	0.89 ***

*** indicates *p* < 0.001, ** indicates *p* < 0.01.

## References

[1] Mobility after Stroke. http://www.stroke.org/stroke-resources/library/mobility-after-stroke.

[2] Patterson K.K., Parafianowicz I., Danells C.J., Closson V., Verrier M.C., Staines W.R., Black S.E., McIlroy W.E. (2008). Gait asymmetry in community-ambulating stroke survivors. Arch. Phys. Med. Rehabil..

[3] Olney S.J., Richards C. (1996). Hemiparetic gait following stroke. Part I: Characteristics. Gait Posture.

[4] Hsu A.-L., Tang P.-F., Jan M.-H. (2003). Analysis of impairments influencing gait velocity and asymmetry of hemiplegic patients after mild to moderate stroke. Arch. Phys. Med. Rehabil..

[5] Yang S., Zhang J.-T., Novak A.C., Brouwer B., Li Q. (2013). Estimation of spatio-temporal parameters for post-stroke hemiparetic gait using inertial sensors. Gait Posture.

[6] Patterson K.K., Gage W.H., Brooks D., Black S.E., McIlroy W.E. (2010). Evaluation of gait symmetry after stroke: A comparison of current methods and recommendations for standardization. Gait Posture.

[7] Anna A.S., Wickström N., Eklund H., Zügner R., Tranberg R. (2012). Assessment of Gait Symmetry and Gait Normality Using Inertial Sensors: In-Lab and In-Situ Evaluation. Biomedical Engineering Systems and Technologies.

[8] Sung P.S., Danial P. A Kinematic Symmetry Index of Gait Pattern Between Older Adults with and without Low Back Pain. http://journals.lww.com/spinejournal/Abstract/publishahead/A_Kinematic_Symmetry_Index_of_Gait_Pattern_Between.95621.aspx.

[9] Moevus A., Mignotte M., de Guise J.A., Meunier J. (2015). A perceptual map for gait symmetry quantification and pathology detection. Biomed. Eng. OnLine.

[10] Dewar M.E., Judge G. (1980). Temporal asymmetry as a gait quality indicator. Med. Biol. Eng. Comput..

[11] Wüest S., Massé F., Aminian K., Gonzenbach R., de Bruin E.D. (2016). Reliability and validity of the inertial sensor-based Timed “Up and Go” test in individuals affected by stroke. J. Rehabil. Res. Dev..

[12] Hodt-Billington C., Helbostad J.L., Moe-Nilssen R. (2008). Should trunk movement or footfall parameters quantify gait asymmetry in chronic stroke patients?. Gait Posture.

[13] Sant’Anna A., Wickström N. (2010). A Symbol-Based Approach to Gait Analysis from Acceleration Signals: Identification and Detection of Gait Events and a New Measure of Gait Symmetry. IEEE Trans. Inf. Technol. Biomed..

[14] Zhang W., Smuck M., Legault C., Ith M.A., Muaremi A., Aminian K. (2018). Simple Gait Symmetry Measures Based on Foot Angular Velocity: Analysis in Post Stroke Patients. http://embc.embs.org/2018/wp-content/uploads/sites/35/2018/08/99118-EMBC-Final-Program.pdf.

[15] Stroke Impact Scale (SIS). https://www.strokengine.ca/en/assess/sis/.

[16] Kosak M., Smith T. (2005). Comparison of the 2-, 6-, and 12-minute walk tests in patients with stroke. J. Rehabil. Res. Dev..

[17] MTw Awinda—Products. https://www.xsens.com/products/mtw-awinda/.

[18] Mariani B., Hoskovec C., Rochat S., Büla C., Penders J., Aminian K. (2010). 3D gait assessment in young and elderly subjects using foot-worn inertial sensors. J. Biomech..

[19] Mariani B., Rouhani H., Crevoisier X., Aminian K. (2013). Quantitative estimation of foot-flat and stance phase of gait using foot-worn inertial sensors. Gait Posture.

[20] Mariani B., Jiménez M.C., Vingerhoets F.J.G., Aminian K. (2013). On-Shoe Wearable Sensors for Gait and Turning Assessment of Patients with Parkinson’s Disease. IEEE Trans. Biomed. Eng..

[21] Mariani B., Rochat S., Büla C.J., Aminian K. (2012). Heel and Toe Clearance Estimation for Gait Analysis Using Wireless Inertial Sensors. IEEE Trans. Biomed. Eng..

[22] Dadashi F., Mariani B., Rochat S., Büla C.J., Santos-Eggimann B., Aminian K. (2013). Gait and Foot Clearance Parameters Obtained Using Shoe-Worn Inertial Sensors in a Large-Population Sample of Older Adults. Sensors.

[23] Moe-Nilssen R., Helbostad J.L. (2004). Estimation of gait cycle characteristics by trunk accelerometry. J. Biomech..

[24] Von Schroeder H.P., Coutts R.D., Lyden P.D., Billings E., Nickel V.L. (1995). Gait parameters following stroke: A practical assessment. J. Rehabil. Res. Dev..

[25] Macbeth G., Razumiejczyk E., Ledesma R.D. (2011). Cliff’s Delta Calculator: A non-parametric effect size program for two groups of observations. Univ. Psychol..

[26] Romano J., Kromrey J.D., Coraggio J., Skowronek J., Devine L. (2006). Appropriate Statistics for Ordinal Level Data: Should We Really Be Using *t*-Test and Cohen’sd for Evaluating Group Differences on the NSSE and Other Surveys?. http://citeseerx.ist.psu.edu/viewdoc/download?doi=10.1.1.595.6157&rep=rep1&type=pdf.

[27] Moufawad el Achkar C., Lenoble-Hoskovec C., Paraschiv-Ionescu A., Major K., Büla C., Aminian K. (2016). Physical Behavior in Older Persons during Daily Life: Insights from Instrumented Shoes. Sensors.

[28] Tan U.-X., Veluvolu K.C., Latt W.T., Shee C.Y., Riviere C.N., Ang W.T. (2009). Estimating Displacement of Periodic Motion with Inertial Sensors. IEEE Sens. J..

[29] Hesse S., Jahnke M., Schreiner C., Mauritz K.-H. (1993). Gait symmetry and functional walking performance in hemiparetic patients prior to and after a 4-week rehabilitation programme. Gait Posture.

